# Single Cell Transcriptomic Analysis of Spinal Dmrt3 Neurons in Zebrafish and Mouse Identifies Distinct Subtypes and Reveal Novel Subpopulations Within the dI6 Domain

**DOI:** 10.3389/fncel.2021.781197

**Published:** 2021-12-23

**Authors:** Ana Belén Iglesias González, Jon E. T. Jakobsson, Jennifer Vieillard, Malin C. Lagerström, Klas Kullander, Henrik Boije

**Affiliations:** Department of Neuroscience, Uppsala University, Uppsala, Sweden

**Keywords:** spinal cord, locomotor network, *dmrt3a*, Wt1a, development

## Abstract

The spinal locomotor network is frequently used for studies into how neuronal circuits are formed and how cellular activity shape behavioral patterns. A population of dI6 interneurons, marked by the Doublesex and mab-3 related transcription factor 3 (Dmrt3), has been shown to participate in the coordination of locomotion and gaits in horses, mice and zebrafish. Analyses of Dmrt3 neurons based on morphology, functionality and the expression of transcription factors have identified different subtypes. Here we analyzed the transcriptomes of individual cells belonging to the Dmrt3 lineage from zebrafish and mice to unravel the molecular code that underlies their subfunctionalization. Indeed, clustering of Dmrt3 neurons based on their gene expression verified known subtypes and revealed novel populations expressing unique markers. Differences in birth order, differential expression of axon guidance genes, neurotransmitters, and their receptors, as well as genes affecting electrophysiological properties, were identified as factors likely underlying diversity. In addition, the comparison between fish and mice populations offers insights into the evolutionary driven subspecialization concomitant with the emergence of limbed locomotion.

## Introduction

Locomotor behaviors are coordinated by subsets of excitatory and inhibitory interneurons in the spinal cord that form a central pattern generator (CPG). These neurons coordinate movements at different locomotor speeds by dictating the recruitment pattern and output frequency of motor neurons (Boije and Kullander, 2018). The locomotor CPG has been widely studied since it allows for analysis of a relatively simple neuronal circuit with a clear functional output. Consequently, several of the transcriptional networks responsible for generating spinal CPG neurons are known (Alaynick et al., 2011; Lu et al., 2015). Twelve progenitor domains have been defined along the dorso-ventral axis of the mouse spinal cord that differentiate into 23 subtypes of neurons (Lu et al., 2015). However, studies of interneurons in mice illustrate the vast heterogeneity that exists within defined populations, as combinatorial antibody labeling revealed at least 50 subtypes within the V1 lineage (Bikoff et al., 2016; Gabitto et al., 2016). To understand how complex motor behaviors are encoded within distinct spinal networks, a thorough description of the heterogeneity within the twelve suggested cardinal classes is needed, as well as linking the subpopulations to their morphological and functional characteristics.

One cardinal class, the dI6 interneurons, have been shown to coordinate gaits and speed transitions in horses, mice and zebrafish, demonstrating their pivotal role within the locomotor network (Andersson et al., 2012; Schnerwitzki et al., 2018; Del Pozo et al., 2020). Mutations in Doublesex and mab-3 related transcription factor 3 (*Dmrt3*), expressed in dI6 neurons, give rise to aberrant locomotor output (Andersson et al., 2012; Del Pozo et al., 2020). Horses carrying a truncating mutation in *Dmrt3* can be trained to develop extra gaits, such as tölt and flying pace in Icelandic horses. Further, null mutant mice show uncoordinated left/right alternation and disturbed flexor/extensor muscle recruitment, whereas mutant zebrafish display aberrant acceleration (Andersson et al., 2012; Del Pozo et al., 2020). Morphological, molecular, and electrophysiological characterizations together suggest that there are functionally distinct subtypes within the dI6 lineage (Andersson et al., 2012; Griener et al., 2017; Schnerwitzki et al., 2018; Perry et al., 2019; Kishore et al., 2020; Satou et al., 2020). The emergence of single cell RNA sequencing (scRNA-seq) has revolutionized our ability to investigate cell type diversity within the nervous system. Several studies have performed transcriptomic analysis of spinal cord cells to describe the differentiation and diversification of the circuitry (Häring et al., 2018; Matson et al., 2018; Rosenberg et al., 2018; Sathyamurthy et al., 2018; Zeisel et al., 2018; Delile et al., 2019; Rayon et al., 2021). A recent study, using droplet based scRNA-seq to characterize mouse neurons, only recovered a few cells originating from the dI6 lineage and excluded this population from much of the following analysis (Delile et al., 2019). Further investigation, using more sensitive scRNA-seq protocols, is thus warranted to characterize this cardinal class of spinal neurons and map their expression profiles to their described subfunctionalities.

Here we used Smart-seq2 to analyze neurons of the Dmrt3 lineage in spinal cords from transgenic zebrafish and mice. We verified subtype specific markers described in mice and identified novel marker genes whose expression defines distinct clusters. An analysis of birth order among the clusters was performed, which could be linked to their physical location within the spinal cord. The expression of ion channels, various receptors for neurotransmitters and axon guidance molecules provide insights regarding functional and morphological differences. In addition, comparison between the two species highlighted evolutionary similarities and differences during development of the dI6 lineage.

## Materials and Methods

### Animals

Adult zebrafish were housed at the Genome Engineering Zebrafish National Facility (SciLifeLab, Uppsala, Sweden) under standard conditions of 14 h light – 10 h dark cycles at 28°C. The transgenic *dmrt3a*:Gal4;UAS:GFP line was previously described (Satou et al., 2013). Zebrafish embryos for the experiments were obtained from group breeding and kept under constant darkness at 28°C. Mice were kept under standard condition of a 12 h day and 12 h night cycle, between 20 and 24°C with constant access to food and water. The mouse line used in this study was Dmrt3*^Cre^*:GT(ROSA)26Sor^TM 14(CAG–tdTomato)Hze^ (Perry et al., 2019; Allen Brain Institute: Madisen et al., 2010), referred to as *Dmrt3^Cre^*:tdTomato along the text. Experiments were approved by a local Swedish ethical board (C 164/14; 5.8.18-11551/19).

### Isolation of Spinal Fluorescently Labeled Cells

For zebrafish, cells were isolated from 3 days post fertilization (dpf) larvae. Using a scalpel, the head (including the hindbrain) and the tip of the tail were removed, and the middle part of the larvae was transferred to an Eppendorf tube. The yolk was removed by gently pipetting up and down in 100 μL of calcium-free Ringer’s solution. Larvae (*n* = 20) were transferred to a protease solution (0.25% trypsin, 1 mM EDTA, pH 8.0, PBS, 50 ml) pre-warmed at 28°C and 27 μL collagenase P in HBSS was added to the samples for 15 min at 28°C with homogenizing every 5 min by gently pipetting up and down. Stop solution (6X, 30% calf serum, 6 mM CaCl_2_, PBS, 10 mL) was added to the tube and spun at 350 RCF for 5 min at 4°C. The resuspension was passed through a 20 μm cell strainer and the sample was placed on ice. Quality control was addressed by comparing live cells versus dead cells using a viability indicator for dead cells (NucRed™ Dead 647 ReadyProbes™ Reagent).

For mice, the spinal cords from three E14.5 embryos were dissected in ice-cold 1X D-PBS (Thermofisher). Both the DRGs and vertebrae column were removed as much as possible. The spinal cords were pooled in one tube and dissociated using the adult brain dissociation kit (Milteny Biotec), adapted for spinal cord tissue. Briefly, 1 ml pre-heated (37°C) enzyme solution 1 (50 μl enzyme P in 950 μl buffer Z) was added to the spinal cords followed by 15 μl enzyme solution 2 (5 μl enzyme A in 10 μl buffer Y). The tissue was incubated at 37°C for 30 min. Every 10 min, the tissue was triturated 10 times with fire polished glass Pasteur pipettes pretreated in 0.5% BSA in 1X D-PBS. The pipets had a decreasing diameter starting with around 200% then 100% and finally around 20% of original opening diameter. Tissue was then transferred to 10 ml ice cold 1X D-PBS and filtered through a 40 μm cell strainer. The suspension was centrifuged at 300 RCF for 10 min at 4°C and the supernatant was discarded. The cell pellet was resuspended in 900 μl 0.5% BSA in 1X D-PBS followed by 100 μl myelin removal beads (Milteny Biotec) and incubated for 15 min at 8°C. 10 ml ice cold 1X D-PBS was added to the sample and centrifuged at 300 RCF for 10 min. Supernatant was discarded and the cell pellet was resuspended in 1 ml 0.5% BSA in 1X D-PBS. The sample was run through an LS-column (Milteny Biotec) on a magnetic stand and flushed with 2 × 1 ml 0.5% BSA in 1X D-PBS. Cell sorting was performed on a BD Melody cell sorter, and tomato positive cells were gated out in comparison to a non-fluorescent control.

### cDNA Synthesis and SMART-seq2 Library Preparation

cDNA synthesis and preamplification were performed using the SMART-seq2 method (Picelli et al., 2014). Briefly, cDNA was transcribed using SuperScript II reverse transcriptase, oligo(dT) primers and template switching oligonucleotides (TSO). Preamplification of the cDNA was facilitated by ISPCR primers that binds the TSO and oligo(dT). The cDNA was tagmented using the Tn5 transposase creating fragments of 200–600 bp, followed by indexation using the Nextera XT sample preparation kits. This was performed by the eukaryotic single cell genomics facility (ESCG) at SciLifeLab, Sweden.

### Alignment and Mapping Reads

Genome indexing and read alignment was performed using STAR v.2.5.4b. Fasta files for extra genes were manually added for eGFP 146 (LC337138.1), tdTomato (GeneBank: AY678269.1) and ERCC genes (Thermofisher assets^1^) and gene information was added to the species GTF file (Shaner et al., 2004; Nakajima et al., 2018). Reads were mapped to GRCm38.99 genome for *mus musculus* or GRCz11.98 genome for *danio rerio*. Genes were counted using featureCounts (subread V2.0.0) and Velocyto v0.17.17 (La Manno et al., 2018). Velocyto spliced and unspliced information was added to the final loom file as separate layers. Full scripts are deposited at Github^2^.

### Pre-processing and Quality Control

To select wells containing viable single cells, several QC metrics were assessed. QC metrics were calculated using scanpy (1.4.4) (calculate_qc_metrics) (Wolf et al., 2018). Mitochondrial genes and ERCC genes were used as quality control variables and were included when calculating QC matrices. We excluded empty wells by only selecting wells with more than 9 or 10 (log1p) total reads for zebrafish and mouse respectively. Cells with biased transcript capture were excluded by removing cells having more than 60 or 40% of all reads in the top 50 expressing genes and less than 1000 or 3000 genes expressed (manually assessed from plots) for zebrafish and mouse, respectively. Furthermore, cells with more than 10% of all reads from ERCC spike-ins or more than 30% (zebrafish) or 20% (mouse) from mitochondrial genes were removed. Total number of reads per cell were normalized (scanpy, normalize_total) to a total of 1 million reads, excluding highly expressed genes (contributing with more than 5% of total reads) (Weinreb et al., 2018). Reads were further log transformed (scanpy, log1p) for downstream analyses.

### Clustering

To detect putative cell identities, principal component analysis was performed using the most highly expressed genes (2000 genes) (Scanpy, highly_variable_genes, pca). A K-nearest neighbor (KNN) graph was constructed (scanpy, neighbors) using 10 neighbors and the Uniform Manifold Approximation and Projection (UMAP) method (Becht et al., 2019). Cell populations were detected using the Leiden community detection algorithm (scanpy, leiden) (Traag et al., 2019) and cell identities were manually annotated using differentially expressed (DE) genes (scanpy, rank_gene_groups) and known marker genes. Clusters were visualized using the UMAP embedding (scanpy, umap).

### Gene Expression

Gene expression was visualized using either UMAP plots (scanpy, pl.umap) or matrix plots (scanpy, matrixplot). Differentially expressing genes among the detected cell identities were based on the group expression compared to the rest of the dataset (scanpy, rank_genes_groups). Here we used the “*t*-test” as method and “benjamini-hochberg” as *p*-value correction method.

### Sub-Clustering

Transcription factors for determining the distinct part of the spinal cord were collected in a list of well-known markers for post-mitotic interneurons in the spinal cord development. We also listed markers for inhibitory and excitatory interneurons.

### Interspecies Subpopulation Correlation

To compare similarities between the subpopulations of zebrafish and mouse, we developed a novel approach to deal with the genome duplication that has occurred in the zebrafish genome. This duplication makes it difficult to find a shared feature space (one-to-one relationship between genes), and it was not possible to use off the shelf integration methods. For each DE gene in one population, we find all orthologous genes in the other species, hence, this could be a one-to-many relationship. Similarity scores were calculated between the populations of the zebrafish and mouse datasets and a similarity matrix was constructed. We assert that DE genes in population ‘‘X’’ represent genes that are less expressed in other populations. Hence, other population that also have high expression of these genes should be consider more similar to population ‘‘X’’ than a population with low expression of these genes. First, the top 10 DE genes for each population in the mouse dataset was determined (scanpy.tl.rank_genes_groups, method = *t*-test), and the orthologous genes in zebrafish was found using Gprofiler^3^. For each cell in the zebrafish dataset, a gene set score was calculated for the orthologous genes using scanpy.tl.score_genes^4^, with 1000 control genes. This was done for each mouse population’s DE genes. The mean score was calculated for all zebrafish populations. We next performed the same calculations from zebrafish to mouse. The results can be represented as individual scores for each cell on the umap embedding or as the average score in a population on an adjacency matrix with source populations as rows, and target populations as columns. Positive score indicated that the DE genes in the source population were expressed higher than a random selection of genes in the target population. Negative scores indicated that these genes had lower expression.

### Inference of Cell Velocities Using Velocyto

Using the dynamics of mRNA transcription and mRNA splicing, information about a cells future transcription state can be inferred (La Manno et al., 2018). For instance, newly initiated transcription of a gene will have high abundance of unspliced mRNA compared to spliced mRNA, as splicing takes time. On the other hand, steady state expression will have a fixed ratio of un/spliced mRNA determined by the transcription kinetics and splicing kinetics. For a recently terminated transcription of gene, there will be high levels of spliced mRNA compared to unspliced mRNA. Here, we used scVelo to model the transcription dynamics for each gene (stochastic model) and from this, we can assess if gene expression is increasing or decreasing in each cell (cell velocities) (Bergen et al., 2020). Investigating the future position of a cell given the expected gene expression changes provides a vector in the embedding indicating the likely transition of the cell. These vectors were plotted on a diffusion map embedding (scanpy.tl.diffmap) as streams (scVelo.pl.velocity_embedding_stream, basis: diffmap) (Haghverdi et al., 2016; Wolf et al., 2018).

### Immunohistochemistry on Zebrafish Tissue

Zebrafish embryos were incubated at 28°C until 3 dpf. Larvae were treated with PTU (0.004%) to prevent pigmentation. Animals were anesthetized with 0.1% of MS-222. The larvae were fixed in 4% paraformaldehyde (PFA) in phosphate buffer saline (PBS) for 15 min at room temperature (RT) and washed in 0.2% PBS-Triton (1xPBS at pH 7.3, 0.2% Triton X-100) three times 10 min. We performed immunohistochemistry in whole mount zebrafish larvae. Larvae were cryoprotected in 30% sucrose in PBS at RT for 2 h. Larvae were equilibrated in 0.2% PBS-Triton (PBS-T) for 10 min three times and treated with acetone for 20 min at −20°C. Larvae were subsequently washed in 0.2% PBS-Triton 2 × 5 min, 2 × 5 min in Milli-Q water and in 0.2% PBS-Triton 2 × 5 min. Non-specific protein binding sites were blocked with 1% BSA in 0.2% PBS-Triton. After the blocking step, larvae were incubated with the primary antibodies in the blocking buffer (mouse anti-Wt1 1:100, Dako; rabbit anti-Calretinin 1:500, Swant) overnight at 4°C in darkness. To remove the residues of primary antibodies, larvae were washed for 2 × 1h in PBS-T. Larvae were incubated with the secondary antibodies (donkey anti-mouse Alexa Fluor 568 1:2000 and donkey anti-rabbit Alexa Fluor 647 1:500, Invitrogen) in the dark at 4°C overnight. Finally, the tissue was rinsed in PBS-T for 2 × 1h and imaged with a confocal Leica SP8 DLS microscope. Quantification of the immunohistochemistry data was performed using the plug-in CellCounter in ImageJ.

### Immunohistochemistry on Mouse Tissue

The vertebrae column from E14.5 *Dmrt3^Cre^*:tdTomato (*n* = 3) were dissected in PBS and fixed for 4 h at 4°C in formaldehyde (4% in PBS, VWR). They were washed three times in PBS and embedded in OCT (Killik Bio-Optica) in cryo-molds (Bio-Optica) using dry-ice cool down isopentane. The spinal cords were sectioned at 20 μm thickness using a cryostat (Cryocut 1800, Leica). The sections were thawed for 1 h and washed twice in PBS before starting the immunohistochemistry. Antigen retrieval was performed by incubating the slides in sodium citrate buffer (0.01M tri-sodium citrate dihydrate and 0.05% Tween-20 pH 6) for 20 min at 85–90°C. The slides were washed twice in PBS before a 1h incubation at RT in blocking buffer (PBS 0.3% triton, 5% donkey serum, and 3% BSA). Then, the slides were incubating in primary antibodies diluted in blocking buffer overnight at 4°C. The day after, the slides were washed three times in PBS before incubation 1 h at RT in secondary antibodies diluted in blocking buffer. Finally, the slides were washed three times in PBS before mounting in Prolong Gold Antifade mounting medium (Thermofisher). The primary antibodies used were mouse anti-Wt1 (1:100, Dako), guinea pig anti-Essrb/NR3B2 (1:2000, gift from Dr. Jay B. Bikoff) and rabbit anti-Prrxl1 (1:500, Rebelo et al., 2007). The secondary antibodies used were donkey anti-guinea pig Alexa Fluor 647 (1:500, Invitrogen A21450) and donkey anti-mouse Alexa Fluor 488 (1:1000, Invitrogen A21202). Pictures were taken using an OlympusBX61WI fluorescent microscope with Volocity software (Quorum Technologies). Image analysis was performed with ImageJ.

For the imaging of whole-mount spinal cord segments from E14.5 *Dmrt3^Cre^*:tdTomato mouse, clearing was performed by incubating the piece of spinal cord in 8% SDS at 37°C for 3–4 h followed by immersion in Iohexol (Omnipaque) overnight at RT. For the whole zebrafish imaging, alive larvae were anesthetized and mounted in 1% low melting agarose. Light-sheet imaging was performed using a Leica SP8 confocal microscope with the DLS module.

## Results

### Transcriptional Profiling of Developing Spinal *dmrt3a/Dmrt3* Expressing Neurons From Zebrafish and Mice

**FIGURE 1:**
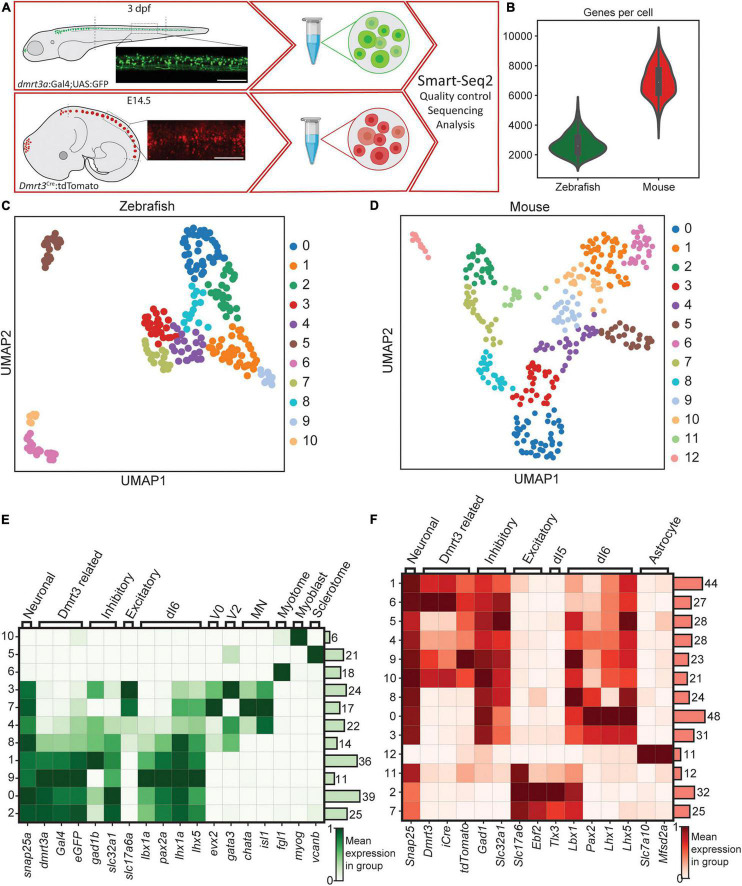
Methodology, the transgenic line, and initial clustering. **(A)** Parts of the spinal cords were collected for zebrafish and mouse. Lateral images depict fluorescent labeling. Tissues were dissociated and scRNA-seq was performed using Smart-Seq2 protocol. **(B)** Number of cells detected per cell for zebrafish (green used throughout figures) and for mouse dataset (red used throughout figures). **(C)** UMAP representation of all cells detected for zebrafish. Individual colors are groups detected by leiden clustering. **(D)** Same as in **(C)**, but for the mouse dataset. **(E,F)** Heatmap of gene expression for the detected leiden groups. Genes were selected to detect different cell types and lineages. Zebrafish dataset is shown in **(E)** and mouse dataset is shown in **(F)**. Color represents the mean gene expression in a particular group of cells. Scale bar in **(A)** equals 100 μm.

Spinal tissue, collected to prevent inclusion of the hind brain, brain stem, and the most caudal part of the spinal cord was used for fluorescence assisted cell sorting (FACS). eGFP positive neurons from *dmrt3a:Gal4;UAS:eGFP* transgenic zebrafish at 3 days post fertilization (dpf) and tdTomato positive neurons from embryonic day 14.5 (E14.5) *Dmrt3*^Cre^*:tdTomato* mice were sorted and collected into 384 well plates (Figure 1A). The selected ages represent similar developmental stages of mouse and zebrafish, corresponding to the end of the initial neurogenic phase in the spinal cord (Sims and Vaughn, 1979; Kimmel et al., 1991; Kulkeaw and Sugiyama, 2012). The cells were sequenced using the Smart-seq2 protocol and reads were aligned to zebrafish and mouse genomes, respectively. After filtering out low quality wells, 233 cells expressing 2699 ± 716 genes (mean ± SD) were obtained for zebrafish and 354 cells expressing 6961 ± 1065 genes for mouse (Figure 1B and Supplementary Figures 1A–H).

### Clustering to Identify Cells Originating From the dI6 Lineage

Initial clustering of the cells identified 11 clusters for zebrafish and 13 clusters for mouse (Figures 1C,D). Using previously described marker genes for various cell types including neurons, oligodendrocytes, Schwann and meningeal cells, astrocytes, vascular cells, oligodendrocyte precursor cells, and microglia cells (Sathyamurthy et al., 2018; Farnsworth et al., 2020), we found that a majority of the clusters were enriched for neuronal markers in both zebrafish and mouse (Figures 1E,F). However, since fine dissection of the spinal cord was not possible in larval zebrafish, multiple cell types were dissociated and sorted. Eight of the zebrafish clusters expressed the neuronal marker *snap25a*, while three clusters (5, 6, and 10) expressed markers for muscle and skeleton cells (Figure 1E). The non-neuronal clusters in zebrafish did not show expression of the reporter *eGFP* or *dmrt3a* and were omitted from further analysis. Neuronal clusters 3, 4, 7, and 8 had weak or no expression of eGFP or *dmrt3a*, expressed both inhibitory and excitatory markers, *evx2*, *gata2a*, *gata3*, *chata*, *isl1*, and *sox1a/sox1b*, indicating a mixture of V0, V2, and motor neurons (Figure 1E and Supplementary Figure 1K). However, since there was some expression of *dmrt3a* and *eGFP* within these clusters, primarily in cluster 8, they were reclustered separately. This led to the identification of an additional *dmrt3a*/dI6 cluster of cells that were retained for downstream analysis (cluster 5, Supplementary Figures 1I,J). Cells in the remaining zebrafish clusters (0, 1, 2, and 9), which had robust expression of *dmrt3a*, *Gal4*, *eGFP*, and expressed markers of the dI6 lineage (*pax2a*, *lhx1a*, *lhx5*, and *lbx1a*), were also retained for downstream analysis (Figure 1E).

In mouse, only cluster 12 expressed non-neuronal markers, which showed characteristics of astrocyte/microglia and lacked expression of *tdTomato* and *Dmrt3* (Figure 1F). Clusters 2, 7, and 11 expressed excitatory markers and were positive for *Pou4f1*, *Tlx3*, *Lmx1b*, and *Prrxl1*, markers for the dI5 lineage (Figure 1F and Supplementary Figure 1L). Since these clusters displayed no or very low expression of *Dmrt3* and *tdTomato* and did not appear to originate from the dI6 lineage, they were omitted from further analysis. The remaining mouse clusters could be divided into two categories, those with strong expression of *Dmrt3*, *tdTomato*, and *iCre* (clusters 1, 4, 5, 6, 9, and 10) and those with weak/no *Dmrt3*, *tdTomato* or *iCre* expression (clusters 0, 3 and 8) (Figure 1F). Even though the second group did not appear to express *Dmrt3*, their expression of *Lbx1*, *Pax2*, *Lhx1*, and *Lhx5*, in the absence of *Ptf1a*, indicate that they belonged to the dI6 lineage and were therefore kept for subsequent analyses (Figure 1F and Supplementary Figure 1L).

The identification of clusters outside of the dI6 lineage, with expression profiles of dI5, V0, V2, and motor neurons, may be due to erroneous capture in the FACS. Cells that sorted into these non-dI6 clusters in fish and mouse were omitted, which left a total of 119 cells from zebrafish and 274 cells from mouse for further analysis.

### Transcription Factors Reveal the Birth Order of dI6 Clusters

During development of the central nervous system in mice, there is a conserved code of transcription factors that reveals the temporal birth order of neuronal subtypes (Delile et al., 2019). Early born spinal neurons (<E10) express *Onecut2*, while neurons born E10-E11 express *Zfhx3, Zfhx4*, and *Pou2f2*, and late-born neurons (>E11.5) express *Neurod2*, *Neurod6*, and *Nfib* (Sagner et al., 2020).

**FIGURE 2:**
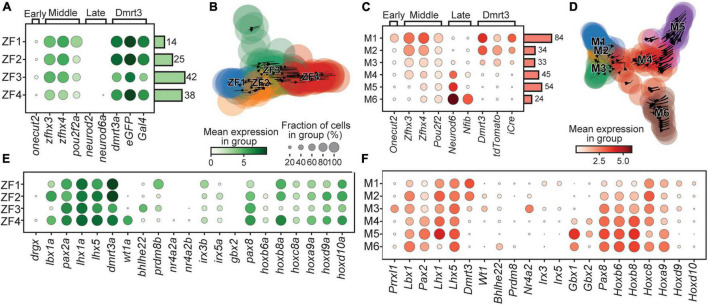
Birth order and rostro-caudal origin of the clusters. Dotplot of genes associated with the time of birth for zebrafish **(A)** and mouse **(C)**. Stream plots of all cells in the zebrafish **(B)** and the mouse **(D)** datasets plotted using diffusion map embedding. Arrows in the plot represents the average velocity of cells in that region. Given time, cells would follow the arrows. **(E)** Dotplot of transcription factors and lineage associated genes in zebrafish. **(F)** Same as **(E)** but for mouse. In dotplots, size of dots represents the proportion of cells in a group that has transcripts for a given gene and the color intensity represents the mean expression level in a group for a given gene.

Cells retained from the zebrafish dataset were re-clustered, resulting in four clusters, named ZF1-4 based on their apparent birth order (Figures 2A,B). The rapid development of zebrafish gave the initial appearance that there was merely a “middle” phase with expression of *zfhx3/4* but not the early or late markers previously described in mice. However, differences in expression level of *zfhx3/4* and *pou2f2a* and other genes, such as *pax8*, allowed a preliminary sorting (Figures 2A,E). Then, by analyzing the ratio of unspliced/spliced mRNA, to estimate if gene expression was increasing or decreasing, Velocyto was used to predict cell transitions (La Manno et al., 2018). This flow between cells was used to assess and verify the temporal order of the zebrafish clusters by creating a pseudo timeline (Figure 2B).

The cells retained from the mouse dataset were also re-clustered, using the same parameters as for zebrafish, and named M1–6 based on their apparent birth order. Here, the clusters could be clearly assigned to three time-windows; M1 expressed *Onecut2* indicating an early birth, M2–3 expressed *Zfhx3/4*, suggesting birth during the middle phase, while M4–6 expressed the late phase marker *Neurod6* (Figure 2C). The order was corroborated by the pseudo timeline determined using Velocyto (Figure 2D). The birth order was also correlated to the expression levels of *Dmrt3*, where early born dI6 neurons had high levels while the late born clusters displayed very little expression of *Dmrt3* (Figure 2C). This trend was also seen for the expression of *tdTomato*. The capture of late born cells with low expression of *tdTomato* may be a result of mRNA/protein inherited from dI6 progenitors that have expressed *Dmrt3*, and therefore indirectly *tdTomato*, during the G2-phase, prior to division, providing the cells with sufficient fluorescence to be selected during the sorting. Nevertheless, this fortunate result allowed us to analyze novel subtypes of the dI6 lineage that displayed very low levels of *Dmrt3* expression at the time-point analyzed.

### Expression of Known Lineage Markers and Additional Transcription Factors

A thorough lineage analysis revealed that only a few of the clusters (ZF4, M2, and M3) expressed *wt1a*/*Wt1*, commonly assigned as a dI6 marker (Figures 2E,F; Alaynick et al., 2011; Haque et al., 2018). Similarly, another dI6 marker, *bhlhe22*/*Bhlhe22*, was only expressed in cells belonging to cluster ZF3 and M6 (Alaynick et al., 2011). Interestingly, cells in the M3 cluster expressed *Prrxl1* (also known as *Drg11*), a marker for the dI5 lineage, but did not express other dI5 markers, such as *Tlx3* or *Lmx1b* (Figure 2F and Supplementary Figure 1L; Alaynick et al., 2011). Similarly, cells in the M3 cluster also expressed *Nr4a2* (also known as *Nurr1*), a marker for subpopulations of V0, V1, and V3 cells, however, there was no expression of *Evx1* (V0), *En1* (V1), or *Prox1* (V3) (Figure 2F and Supplementary Figure 1L). In addition, cells in the ZF1 cluster expressed *prdm8b*, a marker for subpopulations of V0, V1, and V2 (Figure 2E and Supplementary Figure 1K). These observations in both zebrafish and mouse, expression of markers associated with the dI5, V0, V1, V2, and V3 populations, reveal that these are not strict markers for a single cardinal class but instead seem to provide a basis for subdifferentiation of multiple cardinal classes, including the dI6 lineage. Additional transcription factors (*irx3b*, *irx5a*, *pax2*/*Pax2*, *pax8*/*Pax8*, *Gbx1*, and *Gbx2*), known to influence the fate of spinal neurons, showed differential expression among the clusters revealing a clear subdivision among the Dmrt3 cells for both zebrafish and mouse (Figures 2E,F). Hox-genes, which are known to provide positional context, also displayed some cluster specificity (Figures 2E,F). *Hoxb6* and *Hoxb8* were DE among the mouse clusters and showed a strong correlation with birth order, where later born cells displayed higher expression levels of both genes (Figure 2F).

### Differentially Expressed Genes Define Subpopulations

**FIGURE 3:**
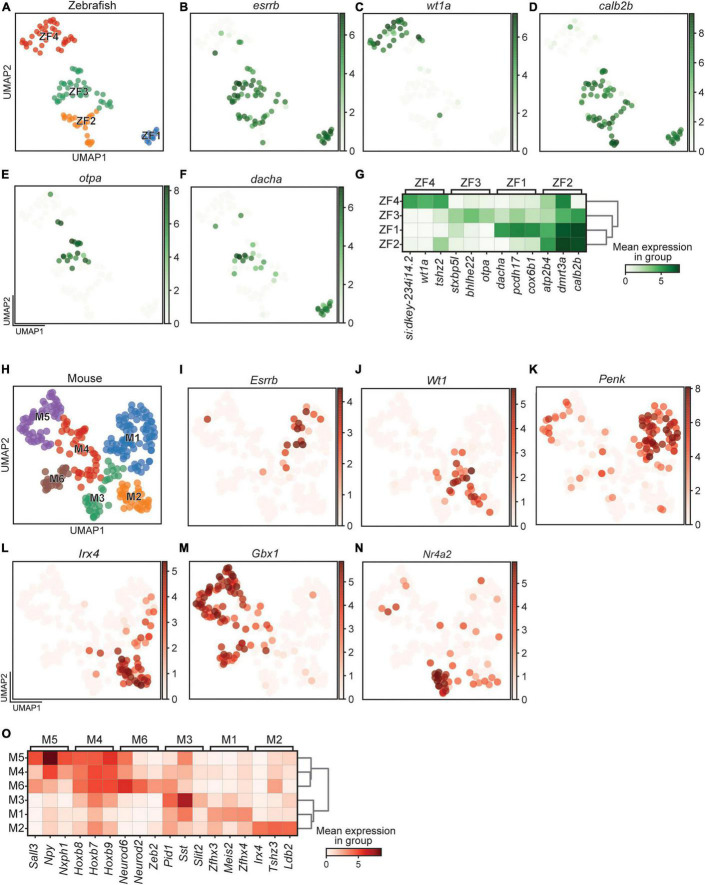
Differentially expressed gene in zebrafish and mouse. **(A)** UMAP representation of all cells retained in the zebrafish dataset. **(B–F)** UMAP representation and expression of individual genes specific to zebrafish clusters. **(G)** Heatmap of the most differentially expressed gene in each zebrafish cluster. **(H)** UMAP representation of all cells retained in the mouse dataset. **(I–N)** UMAP representation and expression of individual genes specific to mouse clusters. **(O)** Heatmap of the most differentially expressed gene in each mouse cluster. Color intensity represents the mean expression level in a group for a given gene.

The clustering parameters were set to identify differently expressed genes in all detected clusters. For zebrafish, this resulted in a split into two major groups, those that expressed *esrrb* (ZF1, 2, and 3) and those that expressed *wt1a* (ZF4) (Figures 3A–C). This expression pattern was largely mutually exclusive for *esrrb* (68 of 119 cells) and *wt1a* (33 of 119 cells), where only five cells expressed both. Also *calb2b* split the four clusters in the same two groups, where *calb2b* expression overlapped with *esrrb* expressing clusters (Figure 3D). Examples of other DE genes, which were used to define the four clusters, were *otpa* for ZF3 and *dacha* for ZF1 (Figures 3E–G and Supplementary Table 1).

Analysis of *Wt1* and *Esrrb* among the 274 mouse cells revealed similarities to zebrafish in that the expression of the two genes was mutually exclusive. However, rather than dividing the clusters in two groups, these two genes were expressed in smaller portions of the cells (Figures 3H–J). Cells that expressed *Esrrb* were found in M1 and *Wt1* expressing cells were present in M2 and M3 (Figures 3I,J). Only four cells were positive for both *Wt1* and *Esrrb*. In mouse, however, the majority of clusters expressed neither *Wt1* nor *Esrrb*. The percentage of cells that were neither *wt1a* nor *esrrb* positive were 82.8% (227 of 274), in contrast to the much lower percentage in zebrafish (19.3%, 23 of 119). Additional DE genes, such as *Penk*, *Irx4*, and *Gbx1*, were the genetic base for the six clusters (Figures 3K–O and Supplementary Table 1). Further sub-clustering would have been possible, as for example seen by the non-overlapping pattern of expression by *Wt1* and *Nr4a2* in the M3 cluster (Figures 3J,N), but the increased resolution needed to divide this cluster led to higher complexity in the downstream analysis, which made the results overwhelming to interpret and convey.

### Immunohistochemical Verification of Subpopulations

**FIGURE 4:**
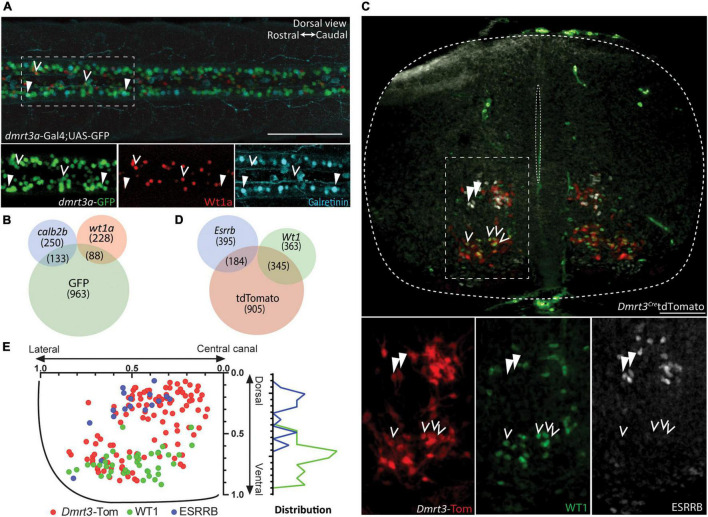
Immunohistochemical verification of subpopulations. **(A)** Dorsal view from double labeling of Wt1a and Calretinin in *dmrt3a*-Gal4;UAS-GFP zebrafish. Images below are magnification of the dashed square. Right: rostral. Left: caudal. **(B)** Venn diagram showing the total number of cells counted, and their overlap. **(C)** Double labeling of WT1 and ESRRB in Dmrt3-Cre/tdTomato mouse. Images below are magnification of the dashed square. **(D)** Venn diagram showing the total number of detected cells, and their overlap. **(E)** Distribution of Dmrt3, Esrrb, and Wt1 along the dorso-ventral and medio-lateral axis in mouse. Scale bar in **(A,C)** equals 100 μm. Arrow heads indicate calretinin, eGFP double positive cells in **(A)** and ESRRB, tdTomato double positive cells in **(B)**, while hollow arrow heads indicate Wt1, eGFP double positive in **(A)** and WT1, tdTomato double positive in **(B)**.

The two distinct non-overlapping populations found in zebrafish, expressing either *esrrb*/*calb2b* or *wt1a*, were selected for immunohistochemical validation. Since none of the tested esrrb antibodies worked on zebrafish tissue, we used *calb2b* and *wt1a* to distinguish and quantify the populations. We performed double whole-mount immunohistochemistry against Wt1a and Calretinin (encoded by *calb2b*) on *dmrt3a:*Gal4;UAS:eGFP transgenic zebrafish at 3 dpf (Figure 4A). Expression of Wt1a was found in 88 of 1184 eGFP positive cells, marking the *dmrt3a* lineage, whereas the calretinin antibody labeled 133 of the eGFP positive cells (Figure 4B and Supplementary Table 2, six segments per fish; five fish). The labeling also revealed that the majority of Wt1a positive cells (228 of 316) and Calretinin positive cells (250 of 383) were not eGFP positive (Figures 4A,B). Five cells co-expressed Wt1a and Calretinin, but none of these were eGFP positive.

Double immunohistochemistry for ESRRB and WT1 was performed on rostral, middle, and caudal sections of spinal cords from three *Dmrt3*^Cre^*:tdTomato* E14.5 mice (Figure 4C). We found that 345 out of 1434 tdTomato cells were positive for WT1 and 184 were labeled for ESRRB (Figure 4D and Supplementary Table 2). Only three cells were double labeled for ESRRB and WT1, but none of these were tdTomato positive. Similar to the zebrafish data, the portion of Wt1 positive cells (363 of 708) and Esrrb positive cells (395 of 579) were not positive for the Dmrt3 lineage (i.e., tdTomato). Birth order has been correlated to soma position in the mouse spinal cord (Hayashi et al., 2018). We therefore registered the location of cells that expressed tdTomato alone, and those that did so in combination with ESRRB or WT1. We found that all three populations had significantly different distribution patterns where the early born ESRRB cells were located more dorsally than the later born WT1 cells (Figure 4E, one-way ANOVA with Dunn’s multiple comparison test). There was no difference in the distribution along the medio-lateral axis (Figure 4E). In addition, overlap between staining for PRRXL1 and tdTomato (54 of 852), which was uniquely expressed in M3, verified this subpopulation within the Dmrt3 lineage (Supplementary Figures 2A,B).

Differences in sensitivity between the two techniques, regulation of translation, mRNA and protein degradation may explain the disparity in the ratio of cells expressing the mRNAs versus the immunohistochemical labeling. Regardless, the immunohistochemical analysis confirmed two distinct populations within the dI6 lineage based on protein expression, in both zebrafish and mouse. Moreover, we found that their somas were differentially situated along the dorso-ventral axis in the mouse spinal cord, where *Esrrb* expressing cells were positioned more dorsally than *Wt1* expressing cells.

### Analysis of Factors Involved in Axon Guidance

**FIGURE 5:**
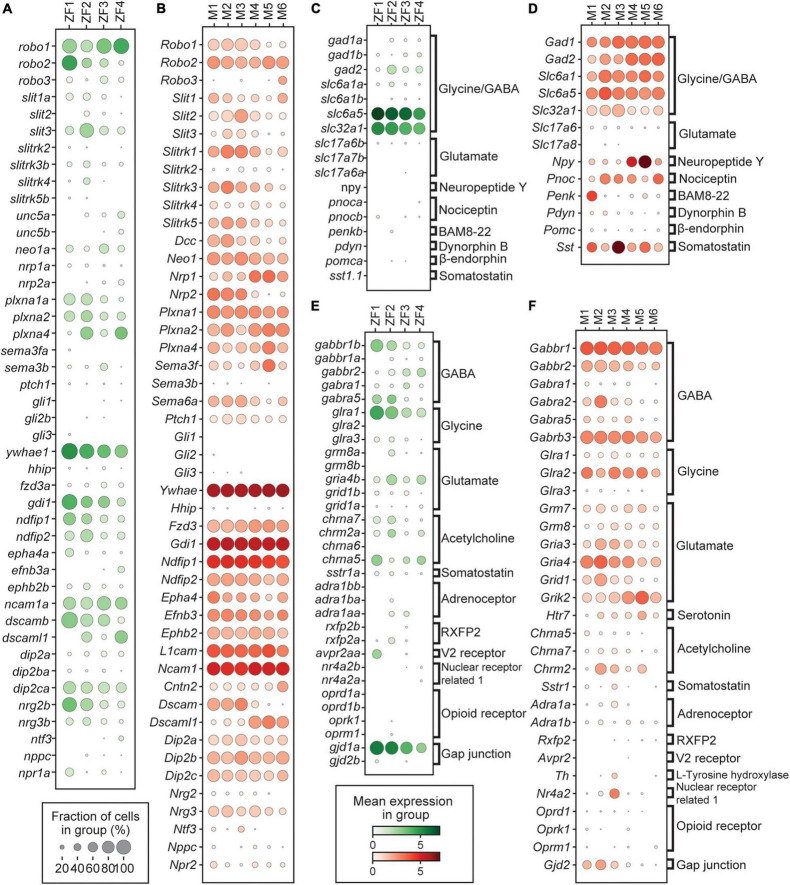
Expression of genes related to axon guidance, neurotransmitters, and their receptors. **(A,B)** Dotplot of genes related to axon guidance for zebrafish and mouse, respectively. **(C,D)** Dotplot of genes related to neurotransmitters for zebrafish and mouse, respectively. **(E,F)** Dotplot of genes related to neurotransmitter receptors for zebrafish and mouse, respectively. In dotplots, size of dots represents the proportion of cells in a group that has transcripts for a given gene and the color represents the mean expression level in a group for a given gene.

To better understand how Dmrt3 expressing neurons are integrated into the locomotor circuitry, we analyzed the expression of receptors and ligands related to axon guidance. We found expression of the slit receptor *robo1*, related to midline crossing (Long et al., 2004), in all zebrafish clusters (Figure 5A). The robo-ligand *slit3* was expressed the most among its family members, displaying a bias for the ZF2 cluster. In mouse, all clusters showed expression of *Robo2* and all but the M5 and M6 clusters expressed *Robo1* while *Robo3* was exclusive to M6 (Figure 5B). The zebrafish clusters also showed some remaining expression of receptors suggesting that they had been sensitive to netrin mediated attraction/repulsion (*unc5a* and *neo1a*) and to semaphorin signaling (*nrp2a*, *plxna1a*, *plxna2*, and *plxna4* with some cluster bias), something that was also reflected in the expression of receptors in mouse (*Dcc*, *Neo1, Plxna1, Plxna2*, and *Plxna4*) (Murakami et al., 2001; Suto et al., 2005; Hill et al., 2012; Figures 5A,B). Whereas zebrafish clusters had limited expression of ephrin receptors and their ligands (*epha4a*, *efnb3a*, *efnb3b*, and *ephb2b*) or semaphorins (*sema3b* and *sema3fa*), mouse clusters showed robust expression of *Epha4*, *Efnb3*, *Ephb2*, *Sema3f*, and *Sema6a*, with some cluster bias. In addition, several other well-known facilitators of axon outgrowth, fasciculation and synaptogenesis were expressed in both species (*dip2ca*/*Dip2c, dscama*/*Dscam, l1cam1a*/*L1cam*, and *ncam1b*/*Ncam1*) (Purohit et al., 2012; Montesinos, 2014; Oo et al., 2020). The ZF1-3 clusters had higher expression of *dscamb* whereas the ZF4 cluster primarily expressed *dscaml1* (Figure 5A). There was a similar bias in mouse where the M1–3 clusters expressed *Dscam* while the M4–6 clusters expressed *Dscaml1* (Figure 5B). Neuregulin-2 and 3 (*nrg2b/Nrg2* and *nrg3b/Nrg3*), involved in neuronal growth and synaptic differentiation, and neurotrophin-3 (*ntf3/Ntf3*), involved in growth and differentiation of new neurons and synapses were also found in the dataset (Figures 5A,B). While *nrg2b* was predominantly expressed in the ZF1 and ZF2 clusters, mouse clusters expressed *Nrg3*. Neither species had clusters that seemed to be responsive to hedgehog signaling as indicated by the absence of expression of the *gli* genes and *hhip*, although mouse clusters showed weak expression of the hedgehog receptor *Ptch1*. Interestingly, all mouse clusters expressed *Fzd3*, associated with Wnt signaling, which was not found in zebrafish (Figures 5A,B). Additional differences found between the two species were *Dcc*, *Sema3f*, *Nrp1*, *Nrp2*, *Ephb2*, and *Epha4*, which were expressed in mouse clusters, whereas the orthologs in the zebrafish clusters showed no or low expression.

### Analysis of Neurotransmitters and Their Receptors

Prediction of neurotransmitter phenotype based on gene expression revealed that all *dmrt3a*/*Dmrt3* clusters, in both species, were inhibitory (Figures 5C,D). In zebrafish, there was some expression of the inhibitory GABAergic marker *gad2* (a glutamic acid decarboxylase involved in neurotransmission) (Soghomonian and Martin, 1998), but robust expression of *slc32a1* (a vesicular GABA and glycine transporter) and *slc6a5* (a glycine transporter), indicating a glycinergic profile. There was no expression of the excitatory markers *slc17a6a* or *slc17a6b* (vesicular glutamate transporters) (Figure 5C). For mouse, the results were similar but expression of *Gad1* and *Gad2* were found in all clusters indicating a dual GABAergic and glycinergic neurotransmitter profile (Figure 5D).

Expression of signaling molecules involved in nociception was minimal in zebrafish, whereas the mouse clusters showed robust expression (Figures 5C,D). Genes encoding endogenous opioid ligands were expressed in mouse; *Penk*, which gives rise to enkephalin and BAM8–22 (bovine adrenal medulla 8–22 peptide) was expressed in the M1 cluster and *Pnoc*, which forms Nociceptin/orphanin FQ (N/OFQ), was expressed in multiple clusters (Figure 5D; Houtani et al., 1996; Abbadie and Pasternak, 2006). There was also strong expression of *Npy* in M4 and M5, which has been associated with both pain and itch sensation (Gao et al., 2018; Jakobsson et al., 2019; Ma et al., 2020; Nelson and Taylor, 2021).

The *dmrt3a*/*Dmrt3* clusters expressed receptors indicating that they respond to inhibitory, excitatory, aminergic, and peptidergic signaling. All clusters in both species expressed ionotropic and metabotropic GABA receptors, but there were some differences regarding the subunits expressed (Figures 5E,F). The same was true for receptors enabling cells to respond to glycine, acetylcholine, and glutamate. Some receptors were selectively expressed in zebrafish, such as the adrenoceptor *adra1aa* in the ZF2–3 clusters and the vasopressin receptor *avpr2aa* in the ZF1 cluster (Figure 5E). There was no significant expression of opioid receptors in either species. In the M3 cluster, *Th* and *Nr4a2*, two dopamine associated genes, were detected and several clusters weakly expressed *htr7a/Htr7*, indicating sensitivity to serotonin (Figure 5F).

In addition to chemical synapses, gap junctions are involved in regulating neuronal activity through electrical synapses, not least in the locomotor network of zebrafish during fast escape responses (Söhl et al., 2005; Satou et al., 2009; Pereda et al., 2013). Analysis of gap junctions (Gj) genes involved in forming electrical synapses, encoding specific types of connexins (Cx), revealed that many Dmrt3 neurons express these gap junction genes (Figures 5E,F). In zebrafish, electrical synapses are asymmetrical where *gjd1a* (*cx34.1)* forms the postsynaptic and *gjd2a (cx35.5)* form the presynaptic part of the gap junction (Miller et al., 2017). While all clusters expressed, *gjd1a*, in decreasing amounts, none expressed *gjd2a* or *gjd2b* (Figure 5E). The mouse ortholog of *gjd1a* and *gjd2a, Gjd2 (Cx36)*, was predominantly expressed in the M1–3 clusters, indicating that these cells may have electrical synapses (Figure 5F).

### Expression of Ion Channels and Other Factors Governing Electrical Properties of Neurons

**FIGURE 6:**
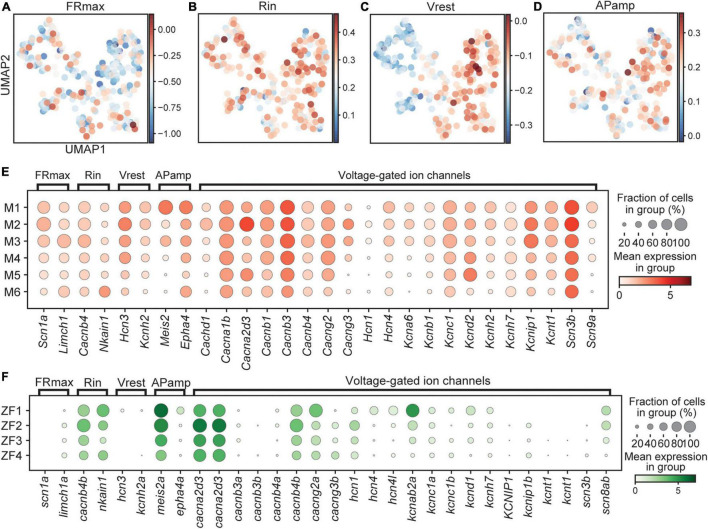
Expression of genes associated with functional properties. Score for genes related to FRmax **(A)**, Rin **(B)**, Vrest **(C)**, and APamp **(D)** in mouse represented on UMAP embedding. Dotplot of genes related to electrophyiological properties in mouse **(E)** and fish **(F)**. In dotplots, size of dots represents the proportion of cells in a group that has transcripts for a given gene and the color represents the mean expression level in a group for a given gene. FRmax, maximum firing rate; Rin, input resistance; Vrest, resting membrane potential; APamp, action potential amplitude.

In addition to receiving external signaling, neuronal activity is also regulated by various ion channels present in the soma and/or axon membrane. A meta-analysis, using transcriptome and electrophysiology data for 34 neuronal types in mice, identified 420 genes whose expression levels correlated with eleven different electrophysiological parameters (Tripathy et al., 2017). From this vast data set, we chose to focus on four parameters; the maximum firing rate (FR_*max*_), the spike amplitude (AP_*amp*_), the resting membrane potential (V_*rest*_), and the input resistance (R_*in*_). While some of the factors that correlated to these parameters were ion channels, directly affecting the membrane potential, others were regulators of ion channels, transcription factors, developmentally regulated genes, or cytoskeletal organization genes. For mouse, we could use the entire dataset to plot a combined score for the genes correlated with a certain trait for each cell. Looking at a population level all clusters anti-correlated with high FR_*max*_, where the difference between M1 and M2 stands out (Figure 6A and Supplementary Figure 3A). There was little cluster-specific correlation regarding R_*in*_, but M1 displayed the highest values (Figure 6B and Supplementary Figure 3B). V_*rest*_ showed distinct correlation bias for M1–3 compared to M4–6, indicating a higher resting membrane potential in early born clusters (Figure 6C and Supplementary Figure 3C). M1 also correlated with higher AP_*amp*_ (Figure 6D and Supplementary Figure 3D).

Singling out genes for illustration purposes, *Scn1a*, encoding the sodium channel Nav1.1, correlate positively with FR_*max*_, was higher expressed in the M1–3 clusters (Figure 6E). Also *Limch1*, with a positive correlation to FR_*max*_, was higher expressed in M3, indicating that early born neurons may be able to fire action potentials at a higher frequency. Expression of *Nkain1*, encoding a protein that interacts with the Na+/K+ pump β-subunit and likely modulates its function, was shown to positively correlate to the input resistance (Armstrong, 2003; Gorokhova et al., 2007; Tripathy et al., 2017). There was higher expression in the M6 cluster and in combination with expression of the anti-correlated gene *Cacnb4*, may indicate a lower input resistance in early born clusters. Regarding V_*rest*_, a positive correlation was previously described for *Hcn3* and *Kcnh2* and we observed higher expression in early born clusters, suggesting that these have a higher resting membrane potential (closer to zero) (Figure 6E). *Meis2* and *Epha4*, which was shown to correlate positively to AP_*amp*_, displayed higher expression in early born clusters, suggesting a higher spike amplitude.

There were clear species differences as genes correlated to FR_*max*_ and V_*rest*_ did not appear to be expressed in zebrafish and thus a full comparison could not be made (Figure 6F). Genes correlated to R_*in*_ and AP_*amp*_ showed mixed results emphasizing the complexity and variability behind these parameters. This is further illustrated by other voltage-gated ion channels, which showed differential expression in genes such as *kcnab2a*, *cacng2a*, and *cacnb4b* for zebrafish and *Cachnd1*, *Cacna2d3*, *Kcna6*, *Kcnd2*, and *Kcnip1* for mouse that affect activation and inactivation kinetics in neurons (Figures 6E,F). However, a deeper characterization of specific genes can provide crucial insights into differences in functional properties.

### Correlation Between Clusters Described in Zebrafish and Mouse

We found a larger heterogeneity among the clusters in mouse compared to zebrafish. To investigate the expansion of the Dmrt3 lineage, we performed relationship comparisons between zebrafish and mouse clusters. There were obvious parallels between the species, revealed by combining birth date and lineage information. In both species, we found that *dmrt3a*/*Dmrt3* expressing neurons were formed first, followed by *dmrt3a*/*Dmrt3* and *wt1a*/*Wt1* double positive cells (Figures 2E,F). However, after the double positive clusters, additional clusters were formed in mouse that expressed no or very low levels of *Dmrt3*.

**FIGURE 7:**
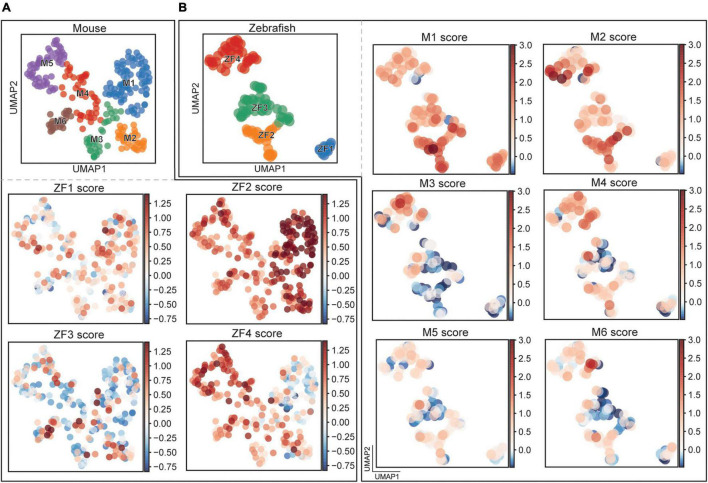
Comparison between mouse and fish clusters. **(A)** UMAP representation of mouse cells with scores for a set of differentially expressed genes found in the zebrafish clusters. **(B)** UMAP representation of zebrafish cells with scores for a set of differentially expressed genes found in the mouse clusters.

By comparing the DE genes in the clusters, a similarity score could be calculated for each cell. Since the number of DE genes varied between the clusters, we compared the top ten genes for each cluster to avoid incorporation of bias. For these ten DE genes, we measured the expression of the orthologous genes in a cluster of the other species and compared this expression to a random sampling of genes within that population. If the expression was higher or lower than the randomly sampled genes, we considered the two populations to be correlated or anti-correlated, respectively, and the link was given a combined score. Going from zebrafish to mouse, we found a temporal correlation where early born zebrafish clusters correlated stronger to early born mouse clusters. The ZF1 cluster got the highest scoring cells in the M1 cluster and the same was true for ZF2, but here also M2 displayed higher correlation (Figure 7A and Supplementary Figures 3E–H). ZF3 had poor correlations to most mouse clusters while ZF4 scored the highest in the M4–6 clusters. For the opposite comparison, mouse to zebrafish, we saw a similar trend, M1 scored higher in ZF1 and ZF2, M2 correlated the strongest to ZF2, while M3 and M4 scored the highest in ZF4 (Figure 7B and Supplementary Figures 3I–N). Once again, the ZF3 cluster seemed the odd one out with little correlation. Interestingly, M5 and M6, born last, showed little similarity to the zebrafish clusters. Taken together, this indicates a conserved progression over developmental time where the additional clusters born late in mouse seem unique to the mouse.

Next, we extracted the genes that underlie the correlation between zebrafish to mouse, and could confirm previously identified similarities such as *Calb2* for ZF2 and *Wt1* for ZF4. We identified new genes of interest, i.e., the transcription factors *Tshz2* and *Tshz3*, which revealed relationships between clusters in the two species. A comparison of mouse clusters to zebrafish clusters verified that temporally regulated transcription factors contribute to their correlation, i.e., *zfhx3/4*, *neurod2*, and *neurod6a/b*. Previously established relationships were also verified for the mouse to fish cluster comparison (i.e., *dmrt3a* and *tshz3a/b*) as well as many new genes indicating close links between clusters in the two species including transcription factors, i.e., *mn1a*/*b*, *meis2a*, *idb2a/b*, *irx4a*, *irx2a*, *skor1a/b*, *gbx1*, and other functional proteins, i.e., *nrp2a/b*, *nell2a/b*, *lingo2a/b*, *nxph1*, and *sez6a/b*. These correlations at the gene expression level suggest that subfunctionalities of the different subtypes may arise from similar gene sets in the two species.

## Discussion

Spinal neurons that express the transcription factor *Dmrt3* during development are involved in coordinating locomotion in vertebrates (Andersson et al., 2012; Del Pozo et al., 2020). These neurons arise from the dI6 domain and characterizations suggest that there are morphologically and functionally distinct subtypes (Griener et al., 2017; Perry et al., 2019; Kishore et al., 2020; Satou et al., 2020). However, a recent transcriptomic analysis of individual spinal neurons was not able to resolve molecularly distinct clusters among the dI6 neurons (Delile et al., 2019). Through single cell analysis of transcriptional profiles of *Dmrt3* expressing neurons in zebrafish and mouse, we have identified differences in gene expression that presumably underlie their subfunctionalization.

### How Does Birth Order and Soma Position Affect the Functionality of Dmrt3 Neurons?

Birth order, soma location, and neurite projections are linked traits that determine a neuron’s role within a network. Temporal mechanisms that underlie neuronal diversification have been described where a transcriptional code can reveal the birthdate of a neuron (Delile et al., 2019). Using the expression of these genes, in combination with Velocyto, we could designate clusters as being born early, mid or late during the formation of both zebrafish and mouse spinal cords (Figures 2A–D). In zebrafish, two phases of spinal development have been described, a primary wave where escape responses and fast swim movements are established and a secondary wave where slower swim movements are established (Myers et al., 1986; Kishore et al., 2014). In a recent article, distinct functional classes were defined among *dmrt3a* expressing neurons in zebrafish; early born (1–2 dpf), consisted of a subtype involved in escape response and a subtype involved in fast swim, and later born (>2 dpf), which were active at slower swims (Kishore et al., 2020). This work allowed us to correlate functional classification and birth-dating with the transcriptional analysis in our study. For zebrafish, an early born cluster (i.e., ZF1) is then more likely to be involved in fast swims while a later born (i.e., the *dmrt3a*, *wt1a* double expressing cluster ZF4) would thus be predicted to be involved in slow swims (Figures 2A,E). Also in mouse, we found that *Wt1* expressing clusters were born after an initial wave of Dmrt3 neurons (Figure 2F), something previously suggested based on a temporal qPCR analysis of *Dmrt3* and *Wt1* in developing mice spinal cords (Perry et al., 2019). Our data thus suggest that birth order for Dmrt3 neurons is conserved between the species and that their birth order and functional properties may be linked. Correlation of cellular activity to frequency output is needed to verify this possibility.

The temporal code has also been shown to divide cardinal neuronal classes into lateral and medial populations (Hayashi et al., 2018). The immunohistochemical labeling of our two temporally distinct populations in mouse, *Esrrb* and *Wt1*, did not detect a difference in position along the medio-lateral axis (Figure 4E). We did, however, see a statistically significant difference in the dorso-ventral distribution, indicating a correlation between birth-date/subpopulation and soma position (Figure 4E). This may also correlate to how these subpopulations interact with motor neurons, where more dorsal motor neuron populations innervate more distal musculature (Machado et al., 2015). Among the different speed-classes in zebrafish, the soma of early born, fast Dmrt3 neurons were ventrally located compared to the more dorsal and medial somas of the later born and slow neurons (Kishore et al., 2020). The dorso-ventral and medio-lateral positioning of the soma is crucial for both the output, local versus projection neurons, and for the input from different types of sensory modalities (Bikoff et al., 2016; Hayashi et al., 2018).

We conclude that different subclusters of the dI6 lineage are born in waves and have distinct soma locations. Further studies are needed to reveal if the different subtypes have divergent synaptic inputs and outputs, which could explain functional differences.

### How Does Morphology Correlate to the Function of Dmrt3 Neurons?

Studies in zebrafish and mouse have concluded that there are morphologically distinct subtypes among the Dmrt3 neurons (Griener et al., 2017; Perry et al., 2019; Kishore et al., 2020; Satou et al., 2020). Three types of Dmrt3 neurons have been described in zebrafish; the early born escape neurons, which sends contralateral local projections (CoLo), the early born fast neurons that have contralateral bifurcating longitudinal projections (CoBL) and the late born slow neurons, which are also CoBL but have a long primary dendrite (Kishore et al., 2020; Satou et al., 2020). Technical differences make morphological characterization more difficult in mice, but analysis of back-filled cells suggest that Dmrt3 neurons send both contralateral and ipsilateral processes that in some cases bifurcate (Perry et al., 2019).

We investigated factors related to axon guidance and regulation of neurite outgrowth that may underlie differences in morphology. The expression of *Robo* receptors, and the netrin receptor *Dcc* in the M1–4 clusters, suggest a tightly regulated neurite outgrowth across the midline. These receptors respond to slit and netrin molecules secreted at the midline of the spinal cord attracting axons, ensuring midline crossing, and subsequently preventing re-crossing (Ypsilanti and Chedotal, 2014). Since these ligand-receptor interactions are involved in multiple processes over time, their exact role in individual neurons is uncertain. Nevertheless, some conclusions can be made, e.g., the expression of *robo1* in all zebrafish clusters makes sense given their contralateral processes (Figure 5A). Interestingly, the early born zebrafish cluster, which likely contains the distinctly shaped CoLo neurons, have higher levels of *robo2* and *gdi1*, factors that may help shape the bifurcation process. Also, the late-born mouse clusters (M5–6) express low levels of *Robo1* and *Dcc*, suggesting that these might represent the ipsilateral populations found in tracing studies.

Interestingly, *EphB2*, *EphA4*, and *EphrinB3* were expressed in mouse but not zebrafish clusters. These axon guidance factors are known be involved in midline crossing in circuits that are important for coordinated gait as well as in selective guidance events for motor axon outgrowth in the limb, suggesting a more diversified role for Dmrt3 neurons in limbed animals (Bonanomi and Pfaff, 2010; Vallstedt and Kullander, 2013).

Additional differences were found, in zebrafish the expression of *plxna4*, *plxna1a*, and *plxna2*, which regulates sensitivity to existing semaphorin gradients, affecting branch pruning, and terminal arborization, differed between clusters. In zebrafish, it was shown that the CoLo, fast and slow *dmrt3a* CoBL neurons, all differed in their innervation of motor neurons. The CoLo neurons, involved in escape, innervate motor neuron axons, fast CoBL neurons made synaptic connections to the soma of medial motor neurons, whereas slow CoBL neurons targeted the dendrites of lateral motor neurons (Kishore et al., 2020). Semaphorin signaling, in combination with ephrins and other factors, may underlie these differences in synaptic connections (Yu and Bargmann, 2001).

It is also noteworthy that the expression of *dscaml1* was found in the late born ZF4 cluster, which likely hosts the slow CoBL neurons with a single long dendrite. Dscaml1 is involved in neurite outgrowth, self-avoidance and avoidance of sister neurites (Montesinos, 2014). Since this is especially important for dendrite tiling, Dscaml1 may provide the means to form non-overlapping arborizations through hemophilic repulsion. This dendrite may form the basis for additional input to the neurons and make them more responsive to coordinate fine movements. This distinction in *Dscaml1* could also be seen among the mouse clusters M4–6, but here there has been no description of dendrites.

We can conclude that axon guidance molecules are diversified in the populations, likely affecting their position within the locomotor circuit. While we can only speculate how varying expression of these factors direct the development of axons, neurites, and terminal innervations, targeted experiments can now be designed. Based on our data, individual factors can be knocked out to fully discern their role in shaping the morphology and synaptic contacts of Dmrt3 neurons.

### Neurotransmitters and Their Receptors Convey Functional Differences

Analysis of genes associated with GABA and glycine signaling revealed that all zebrafish and mouse clusters were inhibitory, which is in line with results previously described (Andersson et al., 2012; Satou et al., 2013, 2020). Analysis of single cells showed that the overwhelming majority of the zebrafish neurons were glycinergic (93.4% *slc6a5/glyt2* positive) while only a portion were GABAergic (42.9% *gad2* positive). The same was true for Dmrt3 neurons in mice regarding the glycinergic profile (91.2%), but a larger portion was also GABAergic (78.1%). The expression of Gad2 was more pronounced in M4–6, a feature that may affect the inhibitory kinetics in the neurons targeted by these later born Dmrt3 neurons in mice (Dumoulin et al., 2001; Xie and Manis, 2014).

Specific gap-junction channels forms fast electrical synapses that are known to be involved in escape responses (Söhl et al., 2005; Pereda et al., 2013; Song et al., 2016). Gap-junction components, *gjd1a* in zebrafish and the mouse ortholog *Gjd2*, were primarily expressed in early born neurons, in line with their involvement in escape responses in fish (Figures 5E,F). In zebrafish, there is one *dmrt3a*-CoLo neuron per segment on each side of the spinal cord that forms a gap-junction with the Mauthner axon, responsible for transferring the escape response along the body of the fish, ensuring mid-cycle inhibition to the contralateral side during the movement (Satou et al., 2009). However, we show that more classes of *dmrt3a* neurons in fish express this gap-junction gene and that it is also present in mouse.

Various endogenic opioids were found to have cluster specific expression in mouse, something that was not observed in zebrafish (Figures 5C,D). Early born mouse cluster M1 expressed *Penk*, which gives rise to enkephalin and BAM8-22 that bind G-protein coupled opioid receptors and modulate nociceptive pain (François et al., 2017). Most mouse clusters expressed *Pnoc*, which produce nociceptin, another neuropeptide involved in pain sensation (Hao et al., 1998). Late born clusters (M4–5) expressed *Npy*, which in the spinal cord has been shown to be involved in pain and itch sensation (Nelson and Taylor, 2021). The endogenous opioids modulate nociception and influence stress response through μ-, δ-, and κ-opioid receptors, which suppress the perception of pain *via* the inhibitory Go and Gi (Al-Hasani and Bruchas, 2011). While expression of such receptors is most abundant in the dorsal horn of the spinal cord, a study showed that the δ-receptor, that preferably bind enkephalin/Bam8-22 and is involved in mechanical pain, was also expressed in V1 inhibitory interneurons (Wang et al., 2018). Specifically, pre-motor Ia interneurons, which mediate inhibition of antagonist muscles, and Renshaw cells, which mediate motor neuron recurrent inhibition, express the δ-receptor, demonstrating a possible connection between pain and the locomotor network (Wang et al., 2018). In mouse, Renshaw cells are known synaptic targets of Dmrt3 neurons and this inhibition of an inhibitory cell may stimulate locomotion during stressful/painful situations (Perry et al., 2019). Indeed, injection of a δ-receptor agonist increased locomotion in rats and a different agonist induced a rapid, transient disturbance of motor coordination with an ataxia-like behavior in mice (Fraser et al., 2000; Jutkiewicz et al., 2005; Gendron et al., 2007; Beaudry et al., 2009). The Dmrt3 neurons in mouse receive direct synaptic input from neurons in the dorsal root ganglia (unpublished observations) and the variance in endogenous opioid used by the Dmrt3 populations suggest differences in their regulatory output. The close developmental heritage between dI6 neurons and the sensory dI4 domain also spurs interest as the M4–5 clusters display intermediate expression profiles, sharing some dI4 associated genes (i.e., *Npy* and *Gbx1*) while lacking others (i.e., *Ptf1a* and *Gsh1/2*) (Figures 2F, 5D). Although much remains unknown, endogenous opioids and NPY in Dmrt3 neurons provide a plausible mechanism linking sensory pain and itch to motor control in mice, a connection that does not seem to exist in zebrafish, which may have arisen alongside limbs.

There were also similarities and differences in the ability of the different populations to respond to neuronal input. All populations in both species carried receptors for GABA, glycine, glutamate, and acetylcholine, however, some variants of receptors/subunits varied in their expression between clusters (Figures 5E,F). Differences in GABA subunits may indicate that the populations have different sensitivity to GABA signaling. Similarly, the early born zebrafish clusters express more glycine receptors, indicating a stronger sensitivity to this type of inhibition. Interestingly, the α1A-adrenoceptors, which binds epinephrine (adrenaline) and is involved in fight-or-flight responses (Wortsman, 2002), was expressed, albeit at low levels, in the ZF2–3 clusters in zebrafish. This is intriguing since it supports the idea that only early born neurons, involved in escape response and fast swims, are sensitive to the adrenaline-rush experienced during dangerous situations. In mouse, we found that the M3 population showed expression of L-tyrosine hydroxylase and Nuclear receptor related 1, two genes associated with dopamine signaling (Sacchetti et al., 2006; Daubner et al., 2011). Dopaminergic neurons are known to modulate the output of the locomotor network and in the case of mouse Dmrt3 neurons this ability seems to be restricted to a single subpopulation (Howe et al., 2019).

In summary, several physiological characteristics diversifies the dI6 population that, depending on the gene expression profile, can be predicted to respond more to sensory input or to modulation of motor control, but also that they likely differ in how sensitive they are to this input. It should be noted that neurotransmitter phenotype and receptor expression have been known to change during development prompting verification of these findings in adult spinal cords.

### Correlation of Transcriptome to Electrophysiological Properties

Besides receptors of neurotransmission, additional ion channels are crucial to determine the firing properties of a neuron. Our general conclusion was that early born clusters in mouse expressed factors indicating that they have a higher firing rate, spike amplitude, resting membrane potential, and lower input resistance (Figure 6). Experiments in mouse did not reveal significant differences regarding these parameters, although, the input resistance tended to be lower in medial and ventral populations (Perry et al., 2019). In zebrafish, there was some correlation as patch-clamping experiments revealed that early born Dmrt3 neurons, active at higher swim frequencies, have a larger spike amplitudes and higher firing frequencies but displayed lower input resistances (Kishore et al., 2020). While electrophysiological properties are complex and interdependent, our transcriptome analysis provides some basis for these features and allow us to correlate gene expression to differences in properties for the different Dmrt3 subtypes. Patch-sequencing experiments are needed to connect gene expression to electrophysiological properties. Also here, our analysis is limited by the developmental stage used as the dynamics of gene expression over time needs to be taken into account and correlated to adult expression and functionality.

### Comparison of Clusters Within and Between Species

A clear split could be seen among the zebrafish *dmrt3a* cells, where expression of *esrrb* or *wt1a* marked a division in a non-overlapping manner (Figures 3B,D). In zebrafish, each segment hosts two CoLo escape neurons, one per hemisegment, and approximately 20 cells belong to the fast and 20 cells belong to the slow CoBL types (Kishore et al., 2020). The four clusters we describe here could potentially provide us with markers for the functional subtypes, but this requires further analysis. Whereas not providing the same clear split, these populations were also found in the mouse dataset (Figures 3I,J). *Wt1* has previously been described as a marker for dI6 interneurons and co-expression of the transcription factors *Dmrt3* and *Wt1* has been demonstrated in the mouse spinal cord (Andersson et al., 2012; Schnerwitzki et al., 2018; Perry et al., 2019). Our immunohistochemical labeling verifies that there exist Dmrt3+/Wt1−, Dmrt3+/Wt1+, and Dmrt3−/Wt1+ cells in both species, demonstrating a level of conservation regarding the development of subpopulations (Figures 4A–D). While only 19.3% of the zebrafish *dmrt3a* cells belonged to the *wt1a* and *esrrb* double negative lineage, 82.8% of the mouse cells do so, revealing an expansion of heterogeneity within the mouse Dmrt3 lineage. Interestingly, we did not identify dmrt3−/wt1+ cells in our transcriptomic dataset despite collecting *Dmrt3* negative populations within the dI6 lineage. This could suggest that cells that only express *Wt1* originate from a different set of dI6 progenitors, which are not part of the Dmrt3 lineage. *Wt1* positive cells, both negative and positive for *Dmrt3*, have previously been characterized as dI6 cells in mouse (Perry et al., 2019). This has not been thoroughly investigated in zebrafish but since all *wt1a* cells are likely to belong to the dI6 lineage, and we confirmed the existence of dmrt3−/wt1+ cells through immunohistochemistry, this adds further unique subpopulations to the dI6 lineage to those identified in our sequencing data.

Having identified conserved subpopulations between the species, we wished to correlate how the clusters were related to each other. There was a clear correlation in timing where early born clusters in zebrafish showed more similarity to early born clusters in mouse and there was an anti-correlation between early/late and late/early clusters (Figures 7A,B). For example, the expression profile of the *wt1a* expressing population ZF4 showed a strong correlation to the *Wt1* expressing clusters M2–3. We also highlighted other factors that are likely responsible for designating subtypes in both fish and mice. It is interesting that the late born zebrafish clusters showed a high correlation to the *Npy* expressing mouse clusters M4–6 that were not found in zebrafish. This could suggest that the lineage has expanded by adding more subpopulations involved in integrating sensory information in the locomotor network as fins have transformed into multi-joint limbs with digits requiring more complex motor control and sensory feedback.

## Conclusion

Through the characterization presented here, we have begun to understand the correlation between birth order and gene expression, and how this governs the soma position, axon innervation, and electrophysiological properties of the dI6 neurons, giving rise to the various subpopulations. Our analysis provides insights to experimentally determined parameters; early born Dmrt3 neurons are ventrally located, active during fast swim, contact the soma of contralateral motor neurons, have low input resistance and high amplitude, to provide a strong mid-cycle inhibition to the opposite side (Andersson et al., 2012; Perry et al., 2019; Del Pozo et al., 2020; Kishore et al., 2020; Satou et al., 2020). Meanwhile, late born Dmrt3 neurons have more dorsal somas, are active during slow swims, contact contralateral dendrites of motor neurons, have higher input resistance and lower amplitude, to provide fine control of movements at slower speeds.

The next crucial step will be to connect soma location, morphology and functionality to the transcriptional profile through patch-sequencing. This would allow us to unequivocally link development and subfunctionality. Further investigations, using anterograde and retrograde tracing to unravel their connectome, is also desired to put their role into a functional context. Our transcriptional characterization of Dmrt3 subpopulations is an important step toward unraveling how subfunctionalities arise and how they are incorporated into neuronal circuits.

## Data Availability Statement

The datasets presented in this study can be found in online repositories. The names of the repository/repositories and accession number(s) can be found below: https://www.ncbi.nlm.nih.gov/geo/query/acc.cgi?acc=GSE185731.

## Ethics Statement

The animal study was reviewed and approved by the Swedish Board of Agriculture *via* the Board of Animal Ethics in Uppsala.

## Author Contributions

HB conceived and designed the study, and wrote the manuscript. KK and ML contributed to conception and design of the study. AI, JJ, and JV performed the experimental work. AI, JJ, and HB performed the data analysis. All authors contributed to manuscript revision, read, and approved the submitted version.

## Conflict of Interest

The authors declare that the research was conducted in the absence of any commercial or financial relationships that could be construed as a potential conflict of interest.

## Publisher’s Note

All claims expressed in this article are solely those of the authors and do not necessarily represent those of their affiliated organizations, or those of the publisher, the editors and the reviewers. Any product that may be evaluated in this article, or claim that may be made by its manufacturer, is not guaranteed or endorsed by the publisher.

## Footnotes

1
https://assets.thermofisher.com/TFS-Assets/LSG/manuals/ERCC92.zip

2
http://github.com/jonetjakobsson/dmrt3-single-cell

3
https://academic.oup.com/nar/article/47/W1/W191/5486750

4
https://www.nature.com/articles/nbt.3192

## Supplementary Material

Supplementary Figure 1 Quality control filtering of datasets and initial heritage assignments of cells. Relates to Figure 1. Threshold used to filter out cells with high fractions of reads in mitochondrial genes **(A,E)**, percent of reads in top 50 expressed genes and number of detected genes **(B,F)**, percent of reads in ERCC genes **(C,G)**, and total number of reads **(D,H)** for zebrafish and mouse respectably. **(I)** UMAP representation of cells selected for further clustering in the zebrafish dataset. **(J)** Heatmap of genes related to Dmrt3 line and cell type identification. **(K)** Dotplot of heritage genes for all groups detected in the zebrafish dataset. **(L)** Dotplot of heritage genes for all groups detected in the mouse dataset. Ptf1a, a marker for dI4, was not plotted in **(J,K)** as no transcripts were identified in the dataset.

Supplementary Figure 2 Relates to Figure 4. Additional immunohistochemical verification of PRRXL1 subpopulation. **(A)** Double labeling of *Dmrt3* and PRRXL1 in Dmrt3-Cre/tdTomato mouse. **(B)** Quantification of *Dmrt3-*Tom, PRRXL1 and double positive cells in Dmrt3-Cre/tdTomato spinal cord samples (*n* = 8).

Supplementary Figure 3 Relates to Figures 6, 7. Cluster averages for correlation analyses. Averages for all mouse clusters regarding electrophysiological parameters; FRmax **(A)**, Rin **(B)**, Vrest **(C)**, and APamp **(D)**. **(E–H)** Average scores for clusters obtained during mouse to zebrafish species comparison. **(I,J)** Average scores for clusters obtained during zebrafish to mouse species comparison. All error bars visualize 95% confidence interval through bootstrapping.

Supplementary Table 1 Relates to Figure 3. Full excel list of genes including differentially expression statistics.

Supplementary Table 2 Relates to Figure 4. Raw data for the immunohistochemical quantification.
