# Supplementary figures and images for: Single Cell Transcriptomic Analysis of Spinal Dmrt3 Neurons in Zebrafish and Mouse Identifies Distinct Subtypes and Reveal Novel Subpopulations Within the dI6 Domain
Source: Front Cell Neurosci. 2021 Dec 23;15:781197. doi: 10.3389/fncel.2021.781197 (PMC8733252; doi:10.3389/fncel.2021.781197)

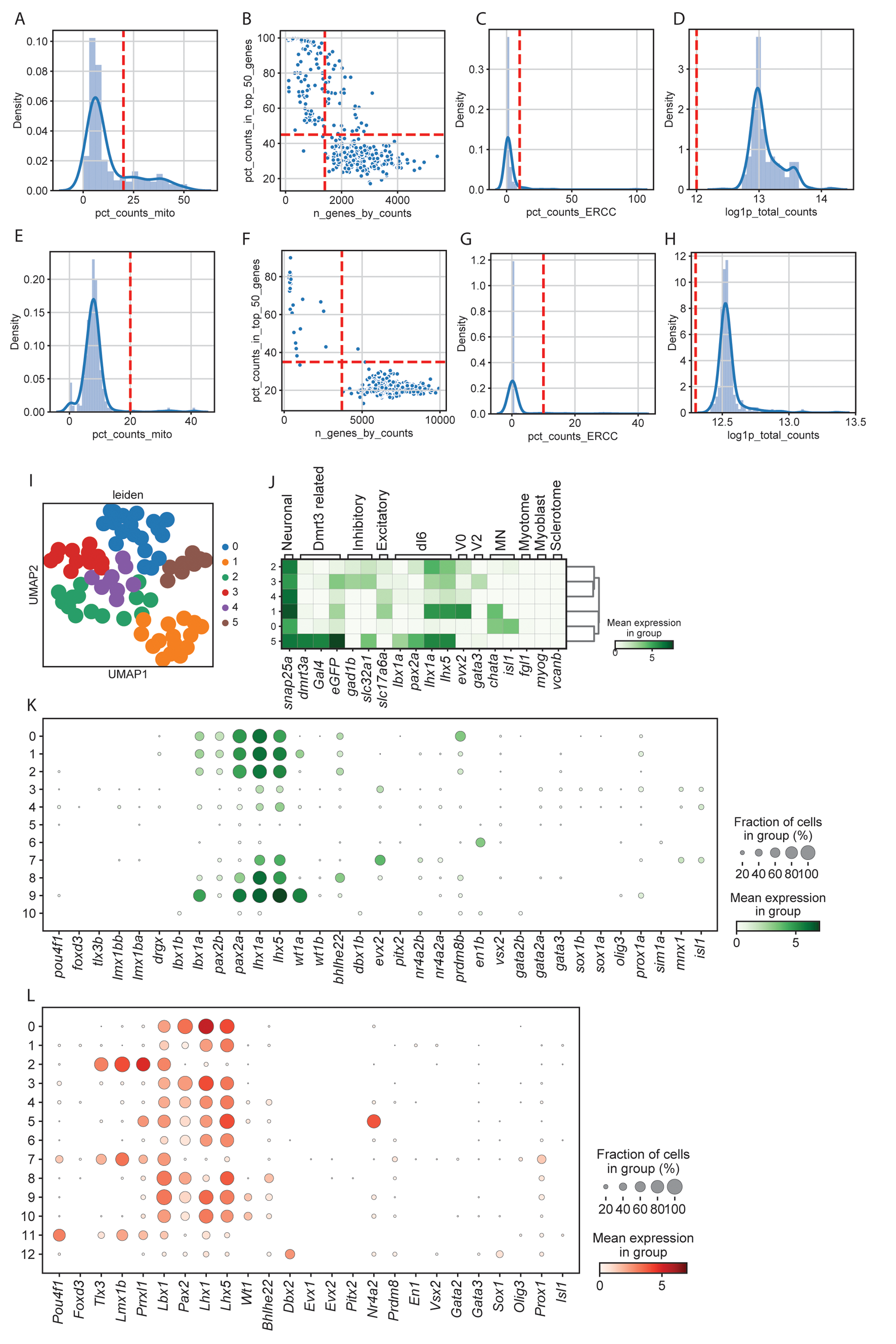

Supplement: Supplementary Figure 1 — Quality control filtering of datasets and initial heritage assignments of cells. Relates to Figure 1. Threshold used to filter out cells with high fractions of reads in mitochondrial genes (A,E), percent of reads in top 50 expressed genes and number of detected genes (B,F), percent of reads in ERCC genes (C,G), and total number of reads (D,H) for zebrafish and mouse respectably. (I) UMAP representation of cells selected for further clustering in the zebrafish dataset. (J) Heatmap of genes related to Dmrt3 line and cell type identification. (K) Dotplot of heritage genes for all groups detected in the zebrafish dataset. (L) Dotplot of heritage genes for all groups detected in the mouse dataset. Ptf1a, a marker for dI4, was not plotted in (J,K) as no transcripts were identified in the dataset. [file Image_1.TIF]

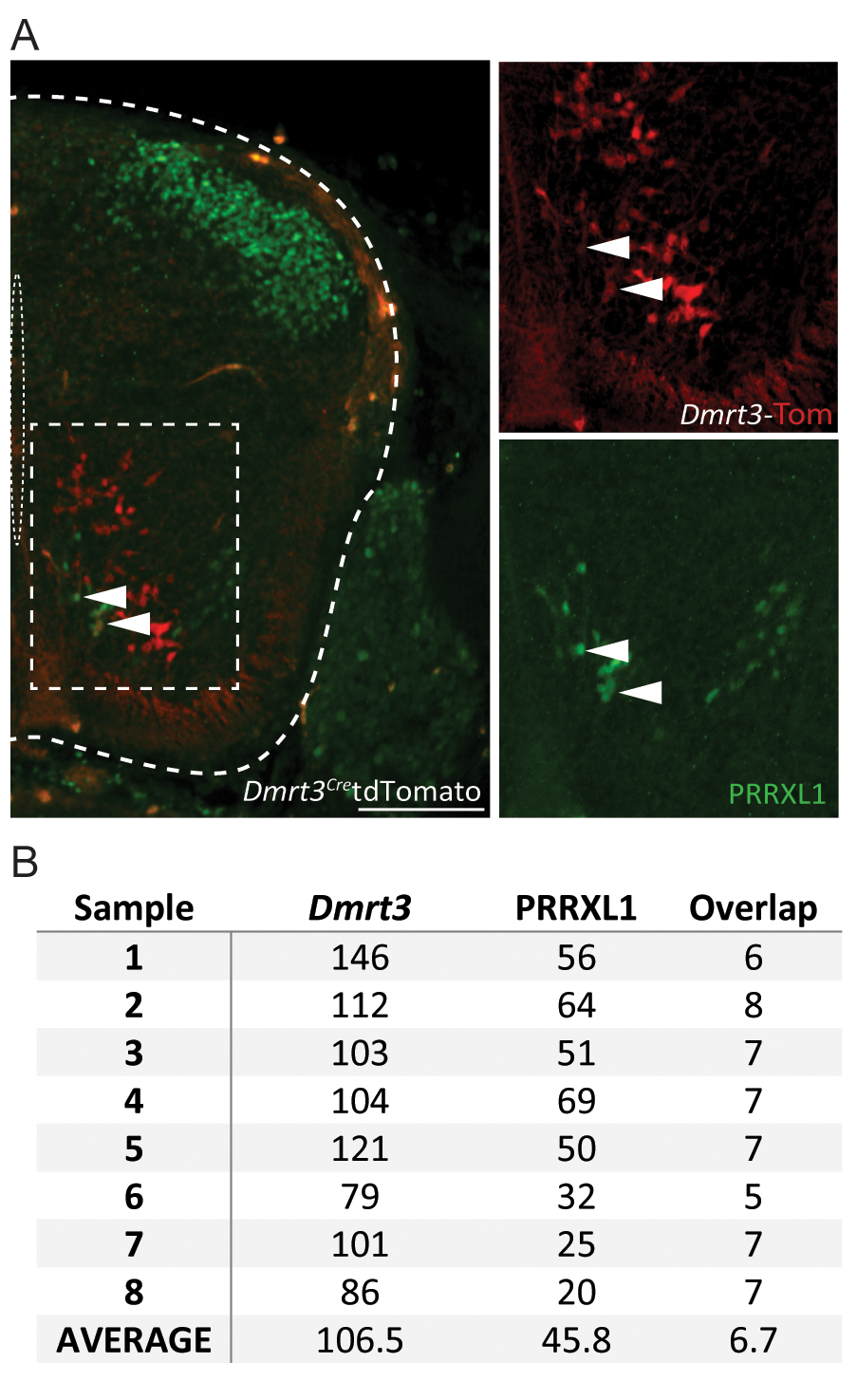

Supplement: Supplementary Figure 2 — Relates to Figure 4. Additional immunohistochemical verification of PRRXL1 subpopulation. (A) Double labeling of Dmrt3 and PRRXL1 in Dmrt3-Cre/tdTomato mouse. (B) Quantification of Dmrt3-Tom, PRRXL1 and double positive cells in Dmrt3-Cre/tdTomato spinal cord samples (n = 8). [file Image_2.TIF]

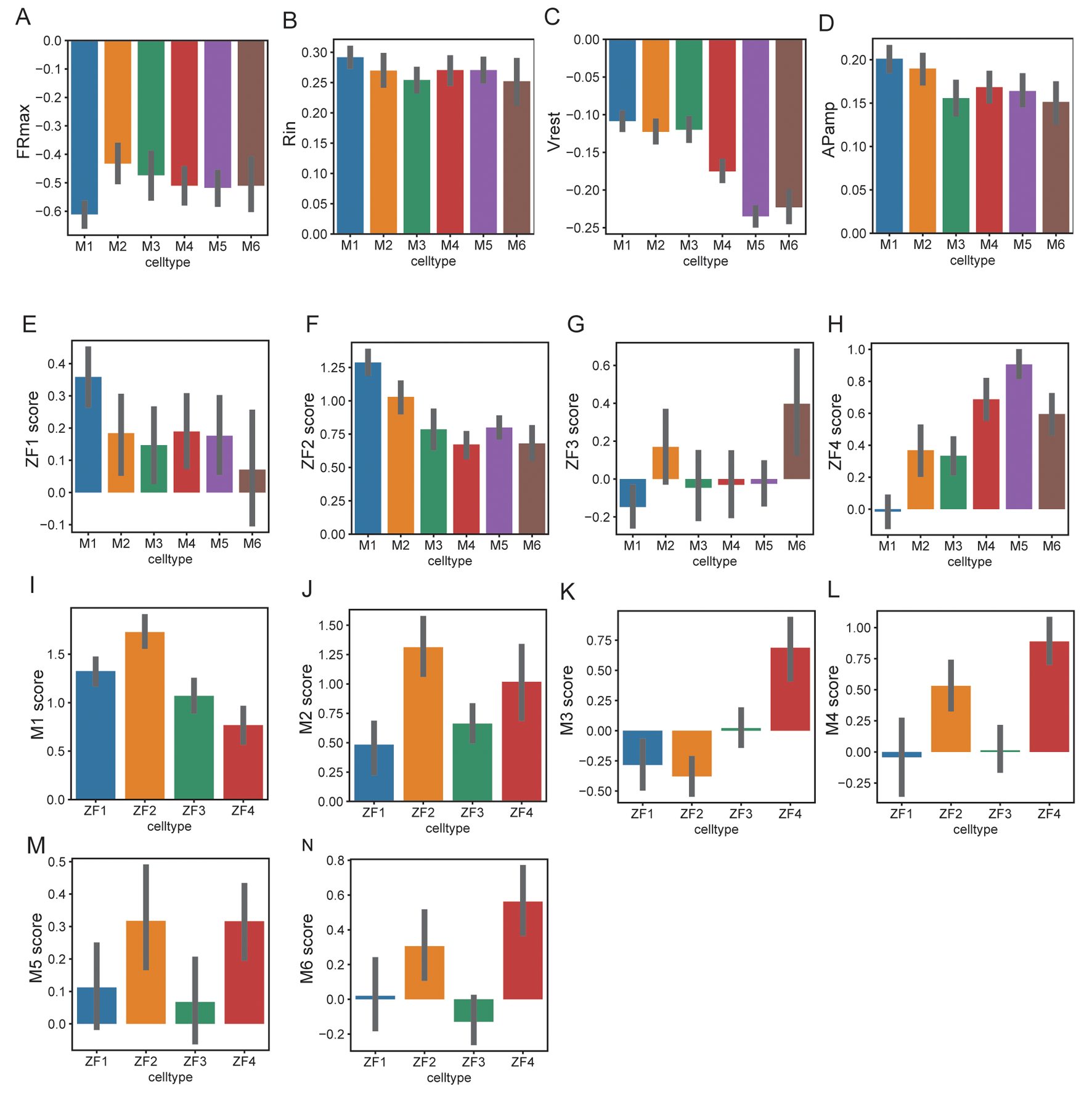

Supplement: Supplementary Figure 3 — Relates to Figures 6, 7. Cluster averages for correlation analyses. Averages for all mouse clusters regarding electrophysiological parameters; FRmax (A), Rin (B), Vrest (C), and APamp (D). (E–H) Average scores for clusters obtained during mouse to zebrafish species comparison. (I,J) Average scores for clusters obtained during zebrafish to mouse species comparison. All error bars visualize 95% confidence interval through bootstrapping. [file Image_3.TIF]
